# Book Reviews

**Published:** 1876-07

**Authors:** 


### Book Reviews.

[NOTE.—All works reviewed in the pages of the CHICAGO MEDICAL JOURNAL AND EXAMINER may be found in the extensive stock of W. B. KEEN, COOKE & Co., whose catalogue of Medical Books will be sent to any address upon request.]

AN ELEMENTARY TREATISE ON DISEASES OF THE SKIN. By *Henry G. Piffard, M.D.*, Professor of Dermatology in the University Medical College. New York and London: McMillan & Co. 1876.

The appearance of a work on Diseases of the Skin written by an American author, even though elementary in scope, may be considered to be an important and significant event in our literature. Perhaps more than any branch of medicine has the one relating to cutaneous pathology and therapeutics been neglected in this country until within a few years past, so that it must be confessed that ten years ago there were few, if any, men capable of writing a systematic treatise on the subject. But within ten years great strides have been made by a goodly number of physicians, chiefly among the younger men, who have studied these affections with a zeal, catholicity and success which has placed them in a favorable light among those recognized abroad as masters in this branch. Indeed, as Americans, we can now proudly claim that our studies and observations on these diseases have been recognized and appreciated in other countries, whose observers are disposed now to look for and avail themselves of the results of our scientific progress in skin diseases, rather than, as in the past, to ignore us, or treat our efforts with slight.

The results of our advance have thus far only been, as we may say, fragmentary, being in the shape of monographs, clinical studies and statistical reports, no one having until now attempted a more or less complete description of the whole subject; yet, though contributions to separate departments of cutaneous medicine, many of them have been of much importance, and it might be well inferred that their authors were fully able to discuss as a whole, that which they only attempted at that time in part. These general ideas occur to us as we take up this excellent little work by Dr. Piffard, an observer who has been a prominent member of the little band which has caused American dermatology to take an honorable stand as a special department of medicine. To those who have read his valuable contributions in the various journals, to those again who have listened to his teaching, and again to those who have watched his painstaking unprejudiced course in the study of dermatology, both clinically and histologically, the fact will be very striking, that in the present instance, a book has been written by a man eminently fitted for the duty. His fugitive essays have shown marked powers of observation and a capacity for the proper generalization and induction from the facts of others—qualities essential to success particularly in such affections as those of the integument. Then, again, owing to the richness of our clinical resources, the author has had an ample field from which to gather his facts. With these general remarks we will now consider the work.

Proceeding to a special consideration of the various subjects of the treatise, we come, first, to the chapter on the anatomy of the skin. Here at the outset, the reader will be favorably impressed with the peculiar characters of the work, namely, conciseness and directness of statement, together with simplicity and clearness of description. We know of no work, certainly none on cutaneous diseases, thus far published, which contains so clear and terse a description of the anatomy of the skin as does the work under consideration. Complex as the structure of this membrane is, and difficult of description within reasonable limits, we find it here presented in a most admirable manner. Another feature of great importance presents itself, one which is observed throughout the entire volume, namely, that of original research, as well as a great familiarity with and judicious employment of the results obtained by others. It is evident that the author has thoroughly familiarized himself with all the various opinions of observers upon the anatomy of the skin, and that he has come to his own conclusions with their aid as well as that of practical study. The whole chapter then can be commended as simple, thorough, and graphic in description.

The illustrations of the structure of the skin and of its appendages are ample in number and apposite in purpose, and much material benefit will be derived from a thorough mastery of the chapter. Following a short and good chapter on the physiology of the skin, we come to that on its pathology, in which the various lesions are described in a terse and simple manner and illustrated by diagrams, which, from their clearness will undoubtedly prove a great help to the student.

The chapter on symptomatology, though containing nothing striking or new, is very satisfactory, and is followed by that on diagnosis, which is, to say the least, excellent in several particulars, as will appear by a short quotation. The author says :

The importance of correct diagnosis in affections of the skin cannot be overestimated; its difficulties, however, have been ; and it is the dread of the supposed intricacies connected with this part of the subject, which deters so many students from embracing the opportunities now afforded them in most of the medical schools of this country. This same feeling, I regret, influences not a few general practitioners, and induces the belief that a useful degree of familiarity with these affections can only be obtained by a prolonged special attention to the subject. I believe that these difficulties have been greatly overrated, and that the student or practitioner who gives a fair proportionate amount of study to this department, will be likely to make fewer incorrect diagnoses than in most of those other branches, with which he feels it his duty to become familiar. Most of the elements upon which a diagnosis in dermatology is to be based, are directly under the eye, while in thoracic, abdominal and pelvic diseases, and in affections of the nervous system, these elements must be sought in deeper recesses, by the aid of physical exploration, or by a most careful analytical study of symptoms, or both. Cutaneous diseases, then, should *prima facie* be easier of diagnosis than others, and in the great majority of instances this is the case. By diagnosis in this connection, I mean the appropriate and correct naming of the affections under consideration.

The author then details the elements of diagnosis, and the sources of information leading thereto, in a manner satisfactory to those already possessing a knowledge of skin diseases and reassuring to those commencing their studies. Besides this, he describes the various appliances auxiliary to diagnosis and their methods of use, thus constituting a noticeable section of his work, one which of all others deserves the attention it has received.

This naturally brings us to the vexed question of classification, and in the chapter devoted to its study we find a succinct description of the merits of the principal one, heretofore used, a consideration of which we cannot enter into here. Suffice it to say, that Dr. Piffard thinks there is room for still another, which, as its basis, depends on the acceptance of the doctrine of certain diatheses as a cause of skin diseases. He divides these diseases into five groups:

I. Diathetic affections.
II. General non-diathetic affections.
III. Reflex affections.
IV. Local affections.
V. Affections of uncertain nature.

He then places the various affections under these heads, of which we cannot here give the full list, and shall merely allude to a few as being more important. By this division, syphilides of course are placed in the diathetic group, and with them are classed the various forms of lupus, which the author, following the French school, and notably Hardy, calls scrofulides. In this connection, we meet with a new name for what has been familiar to us in the writings of the French as the dartrous, herpetic or arthritic diathesis, namely, "the rheumic diathesis," under which pathological head we find eczema, psoriasis and pityriasis. Leprosy is also classed as a diathetic affection, in which class we are somewhat surprised to find icthyosis.

We need say little here of the general non-diathetic affections other than that the group includes principally the eruptive fevers, erysipelas, glanders, etc. Acne is not considered by the author, as by some, a diathetic affection, but rather one of reflex origin, in which group, also, we find rosacea, urticaria, zoster, herpes, and some chloasmata.

Parasitic and non-parasitic affections call for little mention here. The fifth division comprises affections of uncertain nature, which, from its containing such a number of dissimilar troubles is disappointing, and while it shows a weak side of the classification proposed, its perusal carries with it the conviction that a perfect classification of skin diseases is an utter impossibility, at least in the present stage of our knowledge. There are so many points to be considered further on in the book that we refrain here from further criticism of this chapter. It shows great study and care on the part of the author and a laudable desire for simplicity ; and its acceptance or rejection by the reader will largely depend upon his views as to the various diatheses claimed. Of course to a student of the German school it will appear as eminently absurd ; and again, there are points upon which even those who admit diatheses will differ. Our own convictions are such, that we can not unreservedly accept Piffard's classification, still, in a practical point of view it must be confessed that the existence of such is not an absolute essential to a thorough knowledge of these affections, and is useful mostly in giving order and symmetry to the book.

Thus far we have considered what may be called the elementary section, which is comprised in fifty-seven pages, to which fact we call attention, as showing what a mass of knowledge is comprised in so short a space. Turning to the descriptive part we find the chapter on syphilides to be excellent. We commend the skill of the author in presenting this difficult subject so tersely and clearly, and would gladly call attention to several important features. The lesions are described in a graphic manner, and their division is simple and easy of understanding. We are pleased to see the positive statement of the opinions of the author, regarding the necessity of mercury in syphilis, though we must differ from him in attributing to the iodide of potassium greater therapeutic effect than he does. As the author's views upon lupus have already been published, we refrain from comment upon them here; it must in justice to him be added, that this chapter bears the impress of long and patient study, clinically and microscopically. His observations are of great value, and the pronounced views arrived at deserve more than usual attention. Indeed we may well add here, what will be necessary otherwise to often repeat, that the microscopic studies are all carefully made, and that the author has not blindly accepted the work of others, but has, on the contrary, seen most of the appearances described himself. To such a degree is this carried, that the part of this work devoted to microscopy is almost equal to that part in Neumann's great work; but the treatise of Piffard has this great and to us pressing advantage, that clinical features are most clearly brought out, and the microscope is only introduced as an aid to the study of this all important branch. Here we think that the author has shown great judgment, and instead of having written, as did Neumann really, a hand-book of the morbid anatomy of the skin, he has written a thorough clinical treatise in small compass, and has introduced microscopic anatomy only where it will act as an aid and a supplementary study.

In short, we think that one of the most praiseworthy features of this book is the amount of really good original work in cutaneous histology and pathology which it contains, and that another advantage of it is, that this feature has not received undue precedence, to the exclusion of clinical description. In the latter field the author has shown himself a thorough master of his subject, one who has studied minutely and carefully the metamorphoses of the cutaneous lesions. The result is evident, as the clinical descriptions are really excellent in several particulars, namely, terse in statement and graphic in detail, conveying an accurate idea of the lesions as they occur.

Next to clinical description, of course comes treatment, and here again, we have only praise to add. The directions are simple, clear and precise, and the remedies and methods of treatment are, while fully given, much more intelligibly brought out than is usual in books on skin diseases. The few remedies are stated, with directions for their use, and the impression left on the mind is that of certainty, rather than uncertainty. This remark applies to all affections, hence, the matter need not be mentioned again.

We come now to the really cardinal point of the work, namely, its advocacy of a certain diathesis as underlying and causing the various and by far greater number of skin diseases. Whatever may be one's views, no person can read this work without becoming convinced that the author has arrived at his opinions, only after patient investigation of the whole question. He considers the views of the authors who have written on the subject previously, and finally accepts as the underlying cause of the diathesis, which for certain reasons he thinks should be called rheumic rather than dartrous, arthritic or herpetic, a condition of malassimilation or suboxidation, as claimed chiefly by Bence Jones.

This certainly must be said, that the author is not content simply with assuming a diathesis, but that he endeavors to show in what it consists. Much care is shown in the management of this part of the subject, and one reads with interest the remarks of the author on the diathesis itself, and also on its general manifestations.

Eczema and psoriasis are treated of at length, and we may here add that the directions for treatment are simple and practical; indeed, in keeping with the therapeusis of the whole book; they are in the main better, for the reasons given, than in many works which occur to us.

The limits of this review prevent us from calling attention to even a tithe of the interesting features found in the work. On every page we find food for thought and criticism, and can only now refer very briefly to a few.

The chapters on parasitic diseases are excellent, particularly the one on alopecia areata, and in their study, as in other affections, it is evident that the author has worked hard at the clinic and at the microscope. The chapters on keloid, elephantiasis, xanthoma and fibroma molluscum are, as usual, excellent, and in a short space give a clear idea of the present state of our knowledge. This same remark applies to the author's treatment of that curious affection, molluscum contagiosum, in which the latest views are given, criticised and accepted, after personal investigation.

Those interested in the question respecting the similar nature of lichen planus and lichen ruber, will read with interest the author's views, and in the description of them the student will find accurate details.

Such then, is a hasty criticism of a book of much originality; indeed, few volumes have come under our notice in which we have found evidence of so much thorough original work as in this one by Prof. Piffard. For the student, it will prove of great benefit, and we can fully commend it, as it presents the subject of skin diseases uninvested with any obscurity. On the contrary, it presents the necessary knowledge in a plain, simple and eminently clear manner. For the practitioner, it offers the advantages of a clear, concise summary of our present knowledge, which from its directness and terseness of statement will prove a benefit when consulted. The illustrations of pathological growths are for the most part original and excellent, while those showing the anatomy of the skin are duplicates of those from Sappey's celebrated Anatomy, and are perhaps the best ever seen.

The general appearance of the work is strikingly neat, and the type is a marvel of clearness. Finally, we must add that the work reflects great credit upon its author and also upon American dermatological literature. It shows that its author is an ardent student of his subject, and that he is both a careful observer and a cautious thinker.

INSANITY IN ITS MEDICO-LEGAL RELATIONS. By *A. C. Cowperthwait, A.M., M.D.* Philadelphia: J. M. Stoddart & Co. 1876.

This is a monograph in which the author has endeavored to discuss the pathology, classification and diagnosis of insanity, the criminal responsibility of the insane, epileptic insanity, and the treatment of the insane, with an introduction and an index all in the small compass of eighty octavo pages. His first proposition, "that he does not flatter himself that he is bringing forward any strikingly new or original ideas in regard to insanity," we indorse, and are moreover of the opinion that neither in the selections from, nor comments on, his authors has he established the claim that his book contains "the essential facts relating to the pathology and diagnosis and the legal relations of insanity, which should be familiar to every physician, and the knowledge of which is of absolute necessity to him when called upon to testify in courts of justice." We agree with him that the profession at large should pay much more attention than they do to insanity in both its medical and legal relations, not only on account of the increasing prevalence of mental diseases, but also because of the growing tendency to shield under its protection crimes of great enormity; but we doubt very much whether "the wide spread professional apathy and ignorance concerning it" will be much relieved by his contribution to the literature of the subject.

The introduction contains ten rules for the guidance of the physician when called upon to testify as a medical expert. The sixth rule is, "he should not allow himself to be drawn out into giving any opinion on any supposed or imaginary case." If by this he means that the expert should refuse to express an opinion on a hypothetical case, then he shows himself unfamiliar with the rule of practice in many courts.

The second chapter is devoted to the pathology of insanity. Here he quotes extensively from Ray, Maudsley and Schroeder van der Kolk, and enunciates the truism that "diseased mental action is always the product of a diseased brain."

Our author adopts the classification proposed by Dr. Hammond and based upon the division of the mind into the elementary forces of perception, intellect, emotion and will, giving four elementary forms, perceptional, intellectual, emotional and volitional insanity, to which are added mania, characterized by the union of two or more of these forms, general paralysis, idiocy and dementia. We fail to see in what respect this classification is any better than the one proposed by Esquirol thirty years ago.

The fourth chapter is devoted to diagnosis. Here our author justly remarks, "there is no class of diseases so various in their manifestations as those known under the general term of insanity, and their diagnosis is often established with the utmost difficulty," and that "it is only by a practical knowledge gained by experience in the various forms and phases of insanity that this most difficult task is to be accomplished." He moreover tells us that "each individual case must be studied by itself, and nothing but experience and a thorough comprehension of the peculiarities of diseased mental action will prove unfaltering aids."

The next chapter is devoted to criminal responsibility. Upon this important question he makes the following sage remarks: "Should there be a single reason leading to the commission of crime, which is to our perception the offspring of insanity, even though to every appearance the mind is otherwise sound, yet this single delusion or whatever it may be, renders the conscience imperfect, and we are left to decide by the best methods at our command as to the extent of the perversion of the mental faculties by disease, and as to how far the apparently sane faculties sympathize with and are weakened and overcome by those faculties which we know to be diseased. This extremely difficult task is not to be accomplished by establishing any criterion of right or wrong or of legal responsibility of any nature whatever. It must in each individual case be made by the direct examination of scientific men who have studied insanity as a disease and not as a legal problem," and furthermore, "we can not delve within the insane mind and follow its incoherent workings without first becoming insane ourselves, for in this way only could we understand and appreciate its insane reasonings." The practice of common sense and equity and a due consideration of these propositions by the courts would rid the subject of the medico-legal relations of insanity of much of the difficulty that now surrounds it. Our author justly and severely criticises Dr. Hammond for the remarkable positions he has assumed in several cases of public interest.

The next chapter is devoted to epileptic insanity. In this he first briefly notes the symptomatic course of epilepsy, and then enters upon a short discussion of its legal relations. He very correctly states, "that in many instances the epileptic is never fully responsible; a temporary mental derangement being liable to occur subsequent to any mental excitement from fear, anger or any strong moral emotion, and during which a criminal act might be committed." Calling attention to the fact that the epileptic is ever liable to destructive impulses, he justly remarks that "in cases of crime where premeditation and intelligible motives have been absent and unnecessary violence and ferocity used in execution, and especially when in connection with these circumstances there seems to be a partial or complete loss of memory of the act itself, in such cases, the fact of epilepsy being proven, none ought, for a moment, to question the individual's irresponsibility."

The seventh and last chapter is devoted to treatment, and here our author does great injustice to our American Hospitals for the Insane, and gives evidence that notwithstanding "his somewhat extended experience" he is quite unfamiliar with the general principles that govern, and the general results that have been reached in these institutions. This is particularly the case when he makes the remarkable statements that "the practical result of the plans now in operation for the treatment of the insane are in many respects prejudicial to the true interests of the unfortunate sufferers. The crowding together of large numbers of insane persons, of different forms and shades of disease, under one roof, and restraining them there as prisoners behind bolts and bars, to say nothing of the more or less constant cruelty of ignorant and inhuman keepers and attendants, cannot fail to deepen the seeds of disease rather than to bring about a natural and healthy condition of the mental functions." Such expressions cannot be too strongly deprecated, for they not only convey erroneous impressions of the principles that govern the management of our hospitals, but are well calculated to deter friends of the insane from promptly availing themselves of hospital treatment that is frequently so essential to recovery.

Our author proposes that the majority of the cases now in hospital be disposed of by boarding them "two or three together in private and responsible families,"—an arrangement that must appear to almost every one, certainly to all who have had much practical experience in the management of the insane, as very undesirable and quite impracticable. Our author also recommends the adoption, in lieu of our hospitals, of the colony plan of treatment that has been followed at Gheel, in Belgium, for several centuries. This we cannot indorse, because, we do not admit that the plan is successful there, and if successful there, that would be no reason why it would be successful here. The European peasant and the American citizen are entirely different people, and the general character of their insanity is as different as are their other characteristics. If space permitted, we could make other objections to this chapter. D. R. B.

THE ODORLESS WATER CLOSET. A Description of a New Method of Disposing of Sewer Gases. By *R. D. O. Smith*, Washington, D. C. 12 pp., 36 illustrations.

This pamphlet contains a description of a new method of securing perfect ventilation of water closets and soil pipes, which physicians must pronounce the most valuable addition of the age to the sanitary arrangements of modern dwellings. During the past year the medical journals from all parts of this country have contained leading articles on the various diseases arising from sewer gases. In the first week of February, 1876, two of the leading journals of the country, *The Canada Lancet* and *The Medical and Surg. Reporter*, of Philadelphia, started this subject anew. No one is so painfully aware of the prevalence of sewer poison diseases, especially typhoid fever, pneumonia, diphtheria, and infantile diseases, as cosmopolitan physicians of to-day; the object of this notice is to call the attention of just such physicians to the idea contained in this pamphlet.

From the main sewer in the street, a set of tile is laid to the dwelling, and under it, usually to the catch basin. Running perpendicularly from this dwelling sewer to the upper story of the building is the soil pipe. From the sewer main to the top of the soil pipe, there is usually interposed no barrier to the rapid ascent of the extremely light sewer gases. In all water closets heretofore used, water-sealed traps have been relied upon to prevent the escape of sewer gases into the dwelling. But every observer knows that the closets are never free from more or less bad smell. Drs. Fergus and Gleeson, of Glasgow, have recently furnished the explanation of this ever-present smell. According to their carefully repeated experiments the conclusion was reached that sewer gases are transmitted THROUGH the water in a trap in times varying from fifteen minutes to four hours. (*The Sanitary Record*, Dec. 18, 1875, p. 429.)

These gases are never absent from the soil pipe, and their unceasing endeavor is to escape into the dwelling. The idea of the present style of water closets seems to be to force the gases down the soil pipe by dumping a certain amount of water into the trap, and squeezing them out, as it were, into the main sewer. A more absurd method of avoiding the invasion of houses by this deadly foe could not exist. Mr. Smith's pamphlet describes a new departure; instead of *suppressing* or *confining* the foul gases, a free escape is provided for them, and they are conveyed to the top of the dwelling. The escape consists of a pipe leading from the closet to an up-take flue or a chimney. The bowl or receiver of the closet is conical shaped with the apex downwards, and a simple physical law is thus utilized in concentrating the air current and precipitating it into a torrent of sewer gases rising into the ventilator from their own levity. In this way there is a constant draught *down into* the closet, thence through the ventilating shaft to the roof, instead of *up out of* the closet into the dwelling. The advantages of this simple application of physics to the sanitary condition of houses are simply incalculable. This closet can be applied to any place where needed, as residences, hotels, schools, hospitals, prisons, etc. The principle involved in this new idea can be applied to urinals, waste pipes, wash bowls, bath and wash tubs, man holes, sinks and gullies. A movable, odorless commode, connectable with a chimney by means of a flexible tube or a tin tube, can be used in every sick room.

The writer of this brief notice has seen the odorless closet, urinal and commode, and can testify to the absolute absence of any fæcal or urinal odor where they are used. The two former were seen in a place where every traveler can safely say he is usually half strangled by excrementitious stinks before he has half completed his orisons to Cloacinus, viz., in a passenger coach closet; there the air was actually far purer than in the body of the car. A personal inspection of a tenement building in Chicago supplied with this closet, known to the writer, will convince any one of the complete lack of sewer odor about the whole structure.

THE PATHOLOGY AND TREATMENT OF CHILDBED. By *Dr. F. Winckel;* formerly Professor and Director of the Gynecological Clinic of the University of Rostock. Translated from the German, by *Jas. R. Chadwick, M.D.*, Clinical Lecturer on Diseases of Women, Harvard University. 484 pp. Philadelphia: H. C. Lea. 1876. $4.00.

Until within three years, no book has appeared in our language devoted exclusively to the diseases of the puerperal state. During this time, however, the ground has been well occupied by the work of our own countryman, Barker. In view of the fact that the mass of the profession are general practitioners, who are greatly interested in the disorders of childbed, it is not a matter of surprise that the usefulness of another book should be recognized. In the work before us, which we have anticipated with some pleasure, since its announcement more than a year since, there is but little which supersedes the book already alluded to in the field, but much which is confirmatory of, and supplementary to, the teachings of the latter. We learn from the author's preface to the first edition, that the book was first given to the world in 1866; and we might have sooner expected its appearance in English, in this day of numerous translations, for the observations of a man of extended opportunities, like the author's, are always valuable to a great number. In the present instance, a careful study of the book can not fail of being profitable; at the same time, it seems to us, it will do much to elucidate and harmonize many theories of puerperal diseases which are, unfortunately, now vague and discordant.

We have been much interested in the subject matter of the entire book. Besides other topics, we noted especially the author's views of childbed in general: "That childbed exhibits no special conditions" normally, and "that all attempts to demonstrate such conditions must prove futile." This is especially interesting in view of the fact that the majority of English observers have always laid so much stress upon the so-called peculiar condition of a woman in childbed; a condition which favors the occurrence of puerperal disorders in general, and so-called puerperal fever in particular.

Venesection in puerperal diseases has received the attention which the importance of the subject requires. The blood of the pregnant and parturient woman is deficient in the red corpuscular elements, at the same time there is an excess in water, fibrin and white corpuscles. The effect of a general bleeding is to induce just such a condition of the blood, or to aggravate that condition if it already exists. Moreover, venesection is of little efficacy in fever, since it lowers the temperature but a degree or a degree and a half. On the whole, the result of venesection is a speedier loss of strength, and a greater mortality. The effects of local abstractions of blood are often efficient, on the other hand. But in peritoneal inflammation even the latter are best superseded by bags of ice, which should fit the belly like a hood, and which he has never seen freeze the abdominal walls, "even after a week's uninterrupted use" of them.

On page 194, the author says of the use of ice in the treatment of peritoneal inflammation: "As to the use of ice-bags, which I have often employed with women in childbed for more than fourteen days consecutively, and always resort to immediately for all severe pain in the abdomen, when ice is easy to be had, I can only corroborate the statements of Béhier ; I have never seen unfortunate results in my cases, such as diminution of the lochia and lacteal secretion, or affections of the skin, especially gangrene."

The author's consideration of the subject of inversion of the uterus is, at best, incomplete ; no mention being made of Thomas's operation. In this connection it is noticeable that, in the extended bibliography of the author, there is by no means as frequent reference to American literature as in the book of his countryman, Shroeder, who has not overlooked the fact that American gynecologists are really advanced in the science.

We were especially interested in the account of the etiology of so-called puerperal fever, which we believe the most intelligent we have ever read. We infer that the author does not accept a specific disease which can be called puerperal. The affections which may be known as puerperal fever are numerous ; they are usually phlegmasias with the most varied pathology. For clearness, "we must first draw a clear distinction between the first cases of an epidemic and the so called puerperal fever epidemics." "The following have been demonstrated to comprise some of the causes of the affection : (A) In the first place *wounds* and *contusions* of the external and internal genital organs may be succeeded by such inflammations. * * * (B) The *decomposition* of retained portions of the membranes and placenta within the uterus, may also unquestionably evoke all the forms of septic puerperal affections that have been mentioned. * * * (C) Primary inflammations of the vagina and uterus, or gonorrhœa of the genital organs, may become exacerbated in lying-in women to such an extent as to amount to an acute parenchymatous inflammation. * * (D) Finally, it is an established fact, that the most serious puerperal disease may be caused in private practice as well as in hospitals, by the infection of wounded portions of the genital organs with cadaveric matter, or with the secretions of sloughing, or phagedenic wounds." Concerning the beginning of epidemics, the author says: "I have come to the conclusion that puerperal fever originates as an epidemic in lying-in establishments, by means of a direct transfer of the infectious (purulent, putrid or diphtheritic) matter from one puerperal woman to another." Especial attention is called to the fact that the epidemics of puerperal fever, outside of maternities, have been almost exclusively confined to the practice of one man or one midwife.

It may be of interest to the student who desires to keep abreast the fashions in pathology, to know that the disease formerly known as putrescentia uteri, and latterly, metrolymphangitis, is now called diphtheritic inflammation of the uterus and vagina, with thrombosis of the lymphatics and diffuse phlegmon. Alas! the mortality remains the same with all the improvements in nomenclature.

The author does not accept a "milk fever," but refers the elevated temperature which sometimes coincides with the flow of milk, to traumatism or to some phlegmasia.

A feature which enhances the value of the book, is the record of numerous cases interspersed throughout to illustrate the text. In some instances, we fail to see how the conclusions of the writer are warranted by the cases. Thus, on page 328, *et seq.*, a case is reported of death from so-called puerperal fever, metritis phlegmonodes, parametritis, peritonitis, in private practice. Of it he says: "*This case is consequently a proof that in private practice and remote from lying-in establishments, without infection and without miasma, the most severe forms of the septic diseases may occur sporadically in childbed.*" From a careful reading of the record one could hardly conclude that sepsis existed. Moreover, the author himself admits—"there was absolutely no reason for supposing that there was any infection or contagion; much less could a miasmatic origin be suspected. It is, on the whole, most probable, from the early escape of the waters, the long irritation of the cervix, the extraordinary effort made by the uterus to drive the large child into the contracted pelvis, that a metritis with parametritis was set up, and that this caused the peritonitis." On page 201, the case entitled, phlegmonous metritis, paranephritis, peritonitis, pleuritis, pericarditis, continued fever, would, we think, be accepted as an instance of puerperal septicæmia.

An almost universal omission in the records of the cases is the fact of the married or unmarried state of the patient. In most instances perhaps this is not of great importance; but in record No. 47, the omission is a serious one. It may be briefly stated that the writer has reported three distinct forms of mania to which the pregnant and parturient woman is subject: (A) symptomatic mania, accompanying a phlegmasia; (B) mania persecutoria puerperalis; (C) idiopathic puerperal mania. The case alluded to is of the second variety. The subject was 37 years old, of healthy family. When a mere child she received a blow upon the forehead from a flail, not severe enough to destroy consciousness. Her menses appeared at the age of twenty-one; at this time she had a single epileptic attack but never subsequently. She feared her father, who was unkind to her. Becoming pregnant she applied to the Lying-in to make provision for her confinement. During pregnancy her health was good, though at times she was morose. When in labor, she attempted to strangle herself with a towel. The fœtus, dead born, was extracted with the forceps. Post partum, she had puerperal ulcers and endometritis, which grew better under treatment. Gradually, mental derangement became developed, *high fever*, and she soon became the subject of hallucination. Subsequently, abusive and blasphemous language at night. The subject was soon lost sight of, being handed over to the police authorities.

One naturally asks, was this the victim of seduction, and the mother of an illegitimate child? Could her moroseness and attempt at suicide be explained by mortification? In other words, was this not, after all, an instance of true puerperal mania. Writers, especially Barker, have called attention to the fact that seduced and shamed women are the especial subjects of mania puerperalis. Did this woman ultimately recover? The writer places great stress upon the fact of the blow upon the forehead, received many years before, and says that a recovery could not be expected under the circumstances by which the subject was predisposed to brain trouble. There is absolutely no evidence of such predisposition. It is to be regretted that a case so incomplete in detail should be cited in illustration.

In the therapeusis of puerperal mania, it is a little remarkable that the author does not even mention the use of hydrate of chloral.

The translator has done his work so well, that, except for the proper names, we should be but seldom reminded that we were not reading an English book. The centigrade temperature markings have been converted into the Fahrenheit scale. The decimal system of weights and measures has been retained.

Once the meaning of the text was not clear; on page 102, the first two lines of the last paragraph, under the head of anteflexion and anteversion: —— "The vaginal portion is generally pushed backwards, and points towards the sacrum *when the os uteri is inclined forwards.*"

The typographical errors are fewer than is usual in books of this size. On page 224 "mycenium" evidently should be written mycelium. In the last record of the case on page 276, "P. M." should read A. M.

The clear type and general mechanical execution of the book are a source of real pleasure to the reader.

E. W. S.

DETERMINATION OF THE REFRACTION OF THE EYE BY MEANS OF THE OPHTHALMOSCOPE. By *Edward G. Loring, M.D.*, New York. From advance sheets On the Ophthalmoscope. Wm. Wood & Co., New York. 1876. Price, 50 cts.

The perusal of this pamphlet of sixty pages will be a pleasant and useful study for every physician interested in ophthalmic practice. Judging from these advance sheets we believe the whole work promises to be a very valuable addition to American medical literature.

### BOOKS RECEIVED.

REPORT of the State Board of Health of Michigan for 1875.

THE SURGERY, SURGICAL PATHOLOGY AND SURGICAL ANATOMY OF THE FEMALE PELVIC ORGANS, in a series of colored plates taken from nature, with Commentaries, Notes and Cases. By *Henry Savage, M.D.* London. Third edition. Philadelphia : Lindsay & Blakiston. 1876.

TRANSACTIONS of the New York State Eclectic Medical Society for 1875.

AN INTRODUCTION TO PATHOLOGY AND MORBID ANATOMY. By *T. Henry Green, M.D., etc.* Second American edition.

DE LA VALEUR DE L'HYSTEROTOMIE DANS LE TRAITMENT DES TUMEURS DE L'UTERUS. Par Le *Dr. S. Pozzi.* Paris.

### PAMPHLETS RECEIVED.

TRANSACTIONS of the State Medical Society of Kansas, 1875–6.

NOS. 2 and 3, of vol. II, of "AMERICAN CLINICAL LECTURES." On Certain Forms of Morbid Nervous Sensibility. By *J. S. Jewell, M.D., etc.*

THE TREATMENT OF MILD CASES OF MELANCHOLIA AT HOME. By *E. C. Seguin, M.D., etc.*

WARM AND HOT WATER IN SURGERY. A short Historical Sketch, with the present most approved Methods of Application, with Cases. By *Frederick E. Hyde, M.D.*

ON SOME SPECIAL AFFECTIONS OF THE ŒSOPHAGUS. By *Frederick N. Godon, A.B., M.D.*

HYDROADIPSIA AND THE WATER SUPPLY OF LIVING BODIES. By *Z. Collins McElroy, M.D.*

